# Single-visit chairside adjustment of a metal-acrylic resin implant-supported fixed complete dental prosthesis on an unloaded implant using a novel fixed attachment system: a case report

**DOI:** 10.1186/s13256-021-02854-x

**Published:** 2021-05-15

**Authors:** Pedro Molinero-Mourelle, Nadin Al-Haj Husain, Samir Abou-Ayash, Burak Yilmaz, Tateyuki Iizuka, Martin Schimmel

**Affiliations:** 1 Department of Reconstructive Dentistry and Gerodontology. School of Dental Medicine, University of Bern, Bern, Switzerland; 2 Department of Restorative, Preventive and Pediatric Dentistry, School of Dental Medicine, University of Bern, Bern, Switzerland; 3 Division of Restorative and Prosthetic Dentistry, The Ohio State University College of Dentistry, Columbus, OH USA; 4 Department of Cranio-Maxillofacial Surgery, Bern University Hospital, Inselspital, Bern, Switzerland; 5 Department of Reconstructive Dentistry and Gerodontology. School of Dental Medicine, University of Bern, Freiburgstrasse 7, 3010 Bern, Switzerland; 6 Division of Gerodontology and Removable Prosthodontics, University Clinics of Dental Medicine, University of Geneva, Geneva, Switzerland

**Keywords:** Fixed implant-supported prosthesis, Attachment, Biological complication, Technical complication, Case report

## Abstract

**Background:**

Implant-supported prosthetic treatment options are reliable for elderly edentulous patients with systemic health problems. These patients often need cost- and time-efficient solutions to avoid complications. However, it is a challenge for clinicians to treat these patients without surgical interventions, placement of additional implants, or the need to renew existing prostheses.

**Case presentation:**

A 75-year-old medically compromised caucasian male patient using multiple medications was referred for prosthetic rehabilitation of his edentulous maxilla after several implant failures. Because the patient’s health was compromised, further surgical interventions were ruled out and the treatment was centered on the use of the remaining implants by placing a fixed attachment system and altering the existing prosthesis. The stepwise management of the patient’s situation through the use of a new attachment system and adjustment of existing prosthesis is described in the present case report.

**Conclusions:**

Although implant therapy is not always contraindicated for medically compromised patients, it is preferable not to perform extensive surgeries to avoid complications. This clinical report describes an alternative, safe option based on a novel fixed attachment system to salvage an existing maxillary implant-supported fixed complete dental prosthesis of a patient with systemic health problems.

## Background

The increase in worldwide life expectancy has also increased the demand for oral rehabilitation in elderly patient populations. Currently, in Germany and Switzerland, the percentage of patients over the age of 70 has grown considerably. Over 90% of 75-year-olds have been rehabilitated with dental prostheses, and over the past 20 years there has been a surge in implant-supported rehabilitations [1, 2]. This patient group often presents with chronic health issues or polypharmacy, cardiovascular diseases, cancer, respiratory diseases, diabetes mellitus, liver cirrhosis, osteoarthritis, and conditions that involve neurocognitive deterioration [3]. These scenarios must be considered prior to implant therapy, because in some cases the patients or their circumstance can present risks during implant placement, maintenance, and survival [2].

Implant-supported fixed complete dental prostheses (IFCDPs) are considered a predictable treatment option for the prosthetic rehabilitation of edentulous arches. However, biological or technical complications, or a combination of both, such as implant loss may require additional implant placement and/or the fabrication of a new prosthesis [4].

In the case of complications, immediate repair of prostheses is essential for patients with compromised medical conditions to maintain adequate oral function and oral health-related quality of life. However, when the compromised implants are not salvageable, the existing prosthesis may not be readily functional and may require additional implant placement, which might not be possible due to surgical, medical, and/or financial limitations. The fabrication of a new prosthesis may take a long time, which would compromise adequate food intake and can be financially burdensome [5]. In some situations, fewer implants may be used to rehabilitate patients with a functional occlusion (i.e., shortened dental arch) [6–8].

The current worldwide increase in the elderly population along with their associated comorbidities calls for the search for less invasive implant-supported rehabilitation options. A recently introduced attachment system enables the incorporation of an implant that was not included in the existing prosthesis. The use of this attachment system avoids the need for the fabrication of a new prosthesis [5]. However, no clinical studies with long-term follow-up have been published, and to the authors’ knowledge, there are no published reports on the use of this system and technique for the treatment of medically compromised patients.

The aim of this clinical report is to describe a single-visit chairside procedure for the repair and adjustment of an IFCDP following the loss of implants, using a novel angulated attachment system with enhanced angular and rotational freedom in a patient with a compromised medical condition.

## Case presentation

**Fig. 1:**
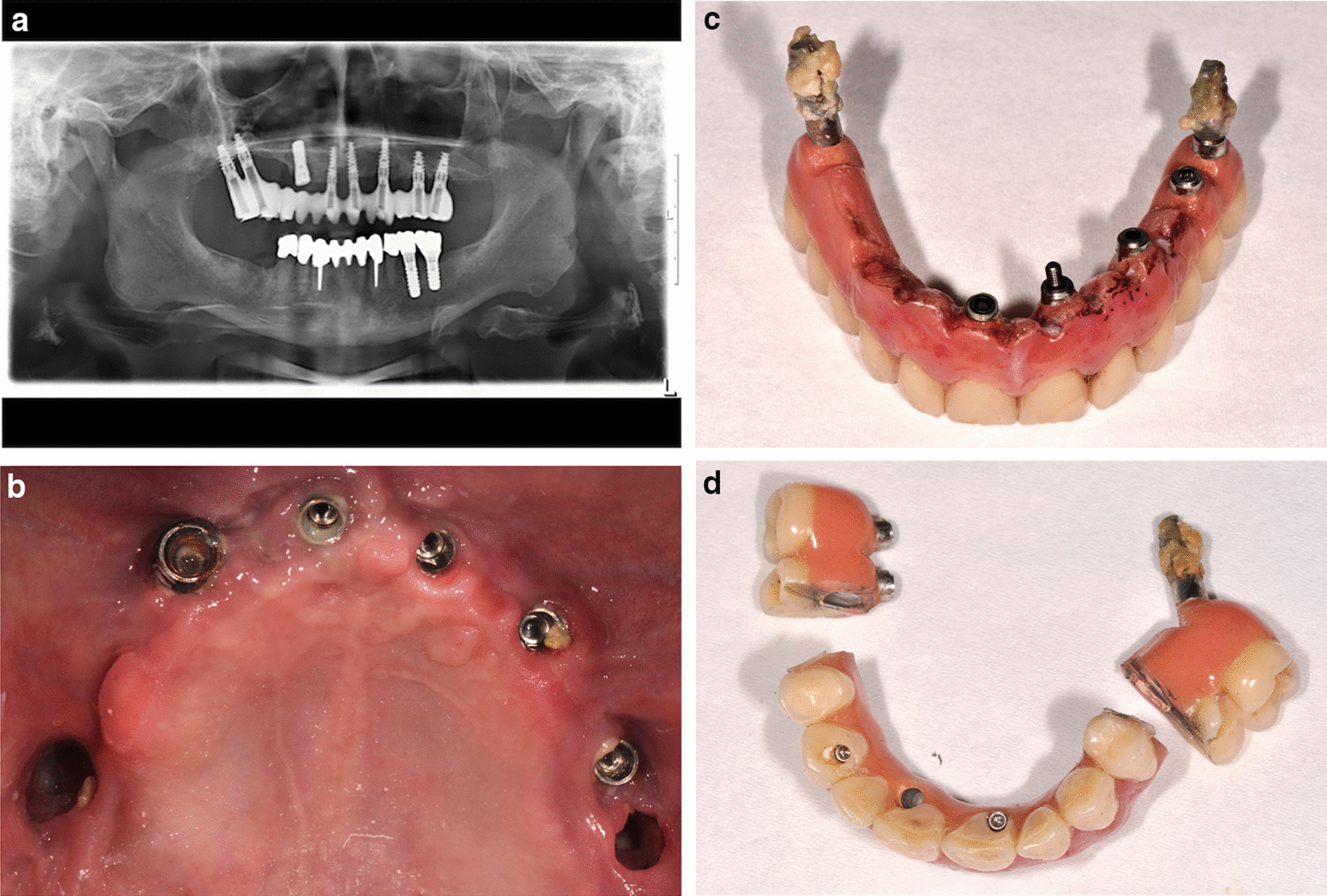
**a** Panoramic radiograph. **b** Initial intraoral situation. **c** Removed prosthesis. **d** New adjustment configuration of the prosthesis

A 75-year-old Caucasian male patient seeking treatment was referred to the School of Dental Medicine, University of Bern, Switzerland. Although the patient presented with a complex medical history involving a lung embolism, heart-acquired valvulopathy treated with an artificial biological valve, advanced diabetes mellitus with paresthesia of the limbs, and high blood pressure, under permanent anticoagulation treatment he was medically stable, being treated and annually reviewed at the Insel Hospital of Bern, Switzerland. The dental history revealed chronic sinusitis that had required several surgical interventions. Eight implants had previously been placed in the edentulous maxilla following bone augmentation procedures including a chin block, tabula externa skull bone, and sinus floor grafting on both sides of the maxilla. The patient was rehabilitated with a screw-retained IFCDP supported by seven of the eight dental implants (NobelActive; Nobel Biocare AB, Göteborg, Sweden) placed in 2010. One of the implants at the right maxillary canine site was not included in the IFCDP (Tapered Effect implant with a regular neck; Straumann, Basel, Switzerland) (Fig. 1a).

The patient had been attending regular follow-up appointments with a dental hygienist since the grafting procedure was performed, and the prosthesis was removed once a year for hygiene purposes. Over the course of 3 years post-prosthesis insertion, a deterioration of the peri-implant tissues became apparent, but the patient did not want surgical treatment of the inflammation due to his general condition and medical issues. He had already lost the implant at the right maxillary central incisor site due to advanced peri-implantitis.

### Pre-prosthetic phase

At the 3-year follow-up appointment, the patient complained of a bad taste in the mouth, discomfort, and leakage of liquid from the mouth to the nose. The radiological examination revealed radiolucency around the posterior implants. In agreement with the patient’s maxillofacial surgeon and otorhinolaryngologist, a decision was made to remove and adapt the patient’s IFCDP to the existing implants prior to the surgical closure of the oroantral communication. Although the patient agreed to the treatment plan, he was not willing either to accept a removable prosthesis as a temporary solution or to leave his IFCDP for adjustments by a laboratory technician. Even though the patient had no financial limitations, he had canceled several appointments for this procedure over the course of a year due to his medical issues.

The dental treatment plan comprised the removal of IFCDP, assessment of the implants and peri-implant tissues, and diagnosis related to the oroantral communication. Because the patient demanded that he receive his fixed prosthesis immediately following the surgical intervention, and depending on the intraoperative findings, it was decided that the IFCDP should be adapted and stabilized with a recently introduced fixed attachment system (Locator F-Tx; Zest Dental Solutions, CA, USA) with the support of the unused implant at the right maxillary canine site. The dental records revealed the brand of the unused implant (Tapered Effect implant with a regular neck; Straumann, Basel, Switzerland), which was placed approximately 15 years ago.

After unscrewing the abutment screws, the IFCDP was removed, and the implant at the right maxillary second molar site came out spontaneously. Additionally, three mobile implants (right maxillary central incisor, left maxillary first premolar, left maxillary first molar) were removed due to complete loss of osseointegration. The three remaining implants (right maxillary canine, left maxillary central incisor and canine) were healthy. The oroantral communication due to peri-implant tissue loss became apparent (Fig. 1b).

### Reconstruction of chairside interim restoration

**Fig. 2:**
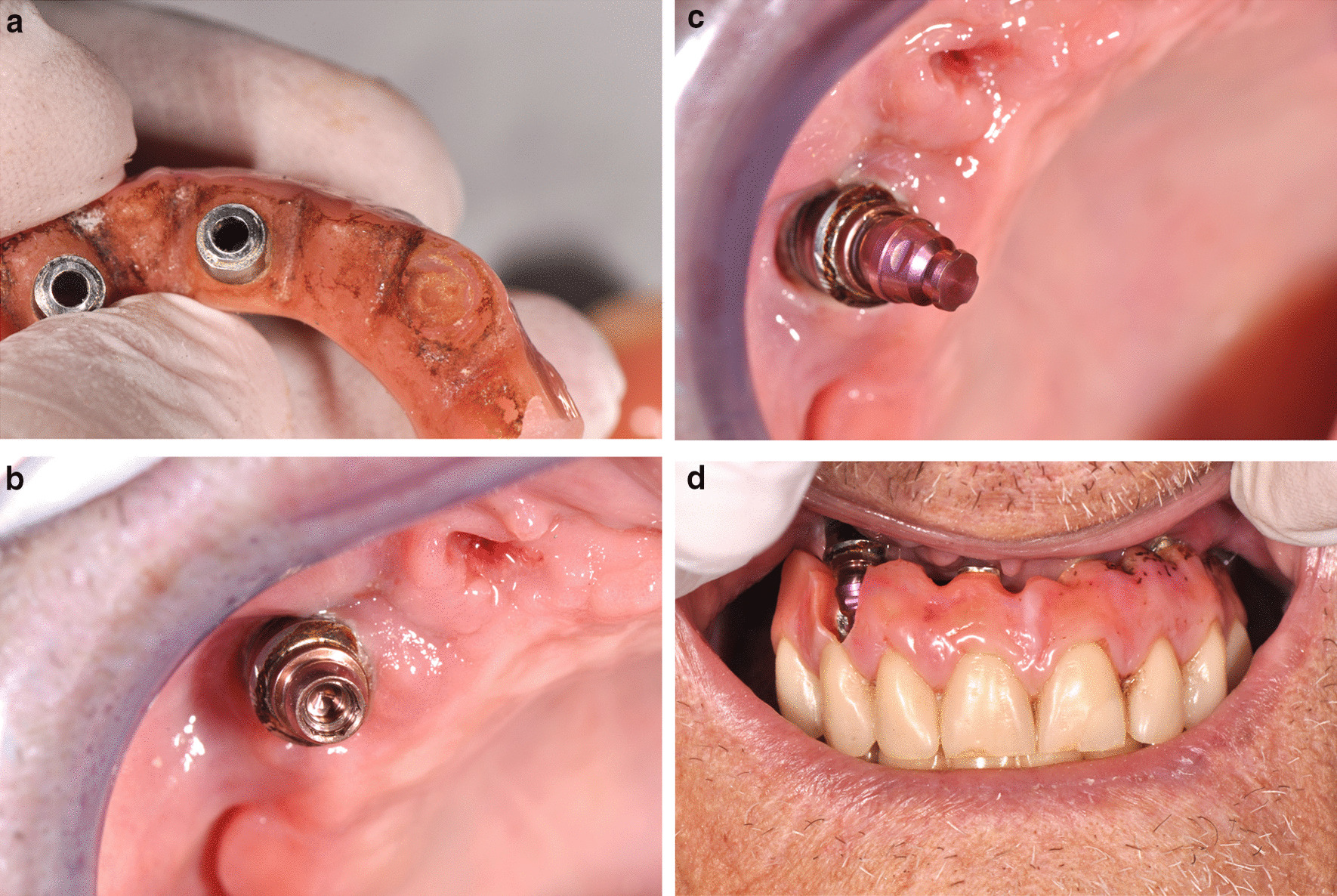
**a** Prosthesis base preparation. **b** Intraoral placement of the abutment of the attachment system. **c** Abutment housing placement. **d** Intraoral try-in of the prosthesis after the attachment system is in place

After cleaning, because the posterior implants were lost, the IFCDP was trimmed distal to the first premolars and the trimmed surfaces were polished (Fig. 1c, d). The previously unused implant at the right canine site was accessible without the need for a surgical intervention, as it was not submerged, and an abutment with an attachment providing angular and rotational freedom (Locator F-Tx; Zest Dental Solutions, CA, USA) was tightened on this implant. The IFCDP was tried in confirming its correct positioning and to confirm whether there was adequate space for the later incorporation of the housing of the attachment system (Fig. 2a). The abutment was then tightened on the implant using a screwdriver and a torque wrench following the manufacturer’s instructions (35 Ncm) (Fig. 2b).

**Fig. 3:**
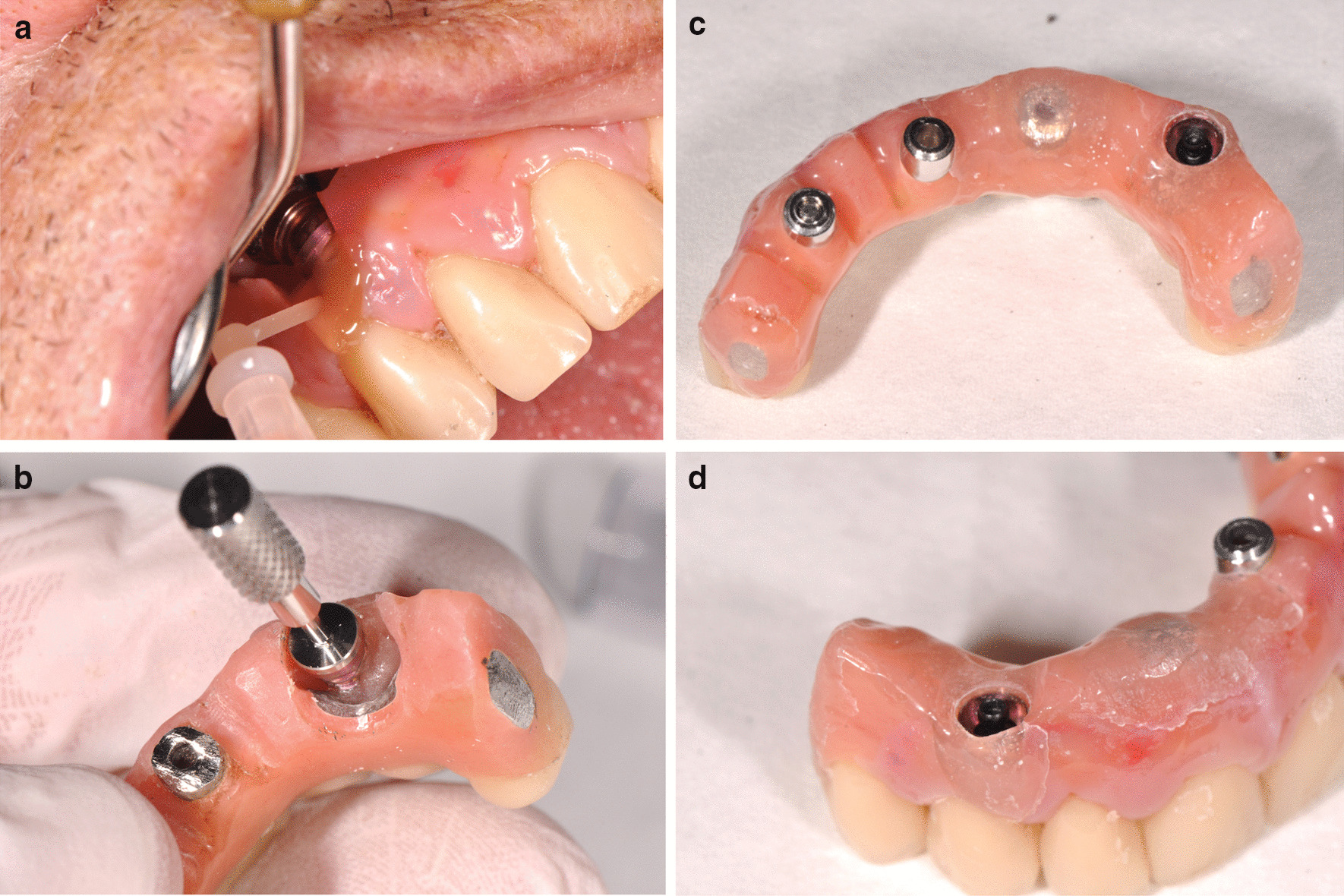
**a** Intraoral relining of the intaglio surface of the prosthesis around the attachment system. **b** Retention ball being placed using the abutment driver. **c** Low-retention ball mounted in the prosthesis. **d** Lateral view of the repaired flange

The IFCDP was gradually adjusted by grinding the acrylic resin using acrylic resin burrs to create sufficient space for the housing, and the acrylic resin surface to receive the matrix was roughened using acrylic resin burrs (Jota AG, Rüthi, Switzerland) (Fig. 2c, d). The abutment housing including the retentive matrix (processing inserts; black) was fixed on the attachment using the abutment driver, and its correct position in the prosthesis was confirmed (Fig. 2d). A block-out spacer was placed between the abutment and the abutment housing. The intaglio surface of the IFCDP to receive the housing was filled with self-polymerizing resin (Chairside Attachment Processing Material; Zest Dental Solutions), and the IFCDP was placed intraorally to incorporate the housing in the prosthesis. Then, the basal and vestibular parts were relined with a self-polymerizing poly(methyl methacrylate) (PMMA) (UNIFAST Trad; GC Corporation, Tokyo, Japan) (Fig. 3a–d). The screws on the remaining implants were tightened. The occlusion was assessed to confirm proper seating. After the processing pick-up resin and PMMA had set, the abutment screws were loosened and the IFCDP was removed. The block-out spacer and the excess resin were removed, and the surfaces of the prosthesis were polished using dental silicone polishing burrs and finished with a polishing wheel (Edenta Exa Cerapol Mounted Grey HP polisher; SG, Switzerland). A universal paste was used for the resin (Renfert GmbH, Hilzingen, Germany).

The IFCDP was fixed on the new retentive abutment, and the abutment screws on the remaining implants were tightened using a torque-controlled screwdriver (35 Ncm). The fit and the occlusion were confirmed, and the screw access holes were first filled with Teflon strips and then composite resin (Telio CS, Ivoclar Vivadent, Schaan, Liechtenstein) (Fig. 3).

**Fig. 4:**
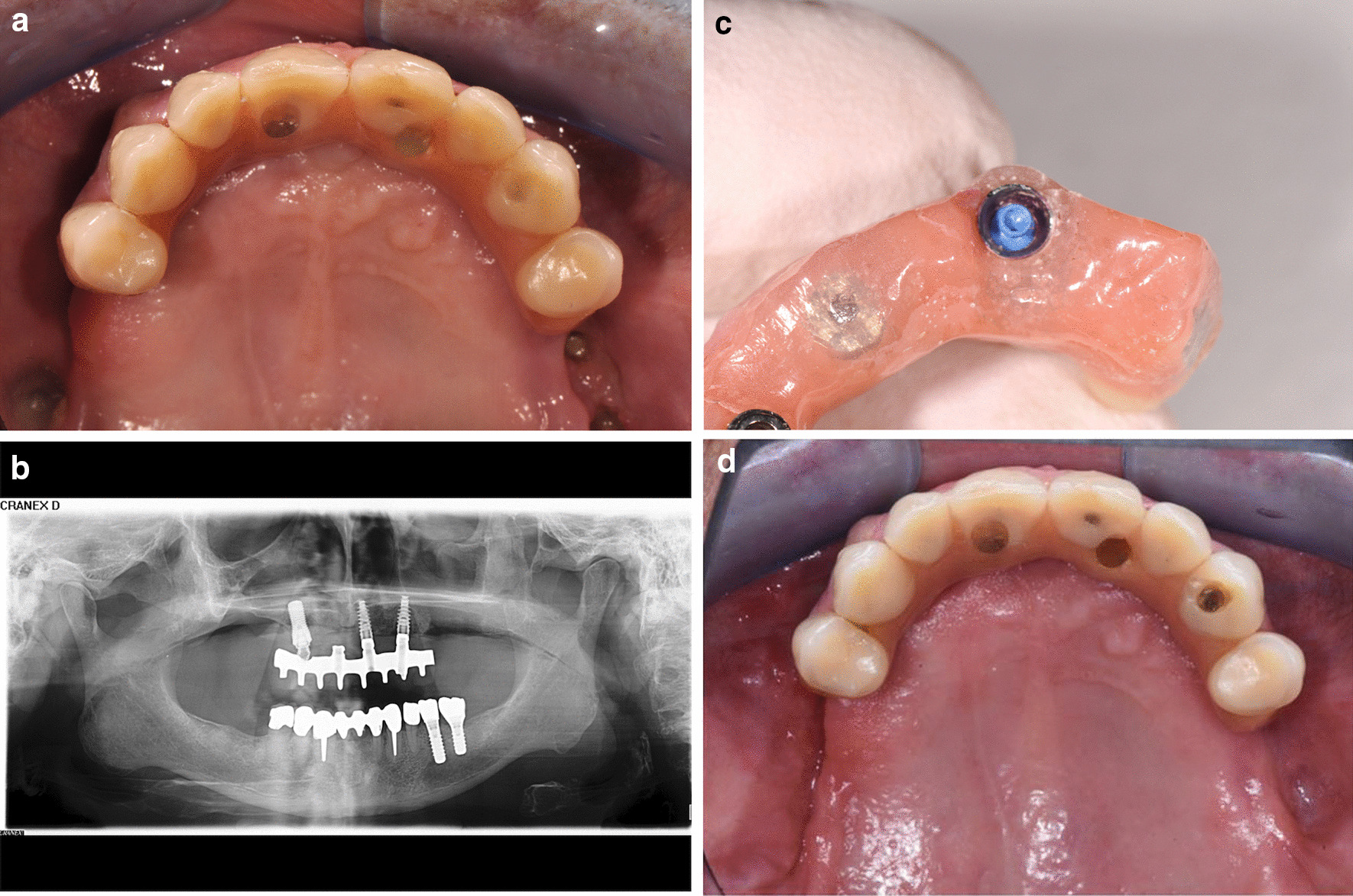
**a** Occlusal view of the new prosthetic design. **b** Panoramic radiograph of the final situation. **c** Mounted low-retention ball attachment. **d** Extraoral frontal view of the final prosthesis

Three months after the delivery of the repaired IFCDP, the patient developed a dialysis-dependent kidney insufficiency. After his kidney-related issue was stabilized by his medical doctors, he underwent surgical closure of the oroantral communications, which included decortication of the osteomyelitic foci in the maxilla and maxillary sinus floor, using intraoral access, and closure of the oroantral communication with a buccal fat pad pedicled flap. The patient was satisfied with the outcome and with the fact that the biological complications were managed by avoiding the need for additional grafting and implant placement (Fig. 4d). Later renewal of the IFCDP was deemed unlikely due to the difficult coordination of dialysis and dental appointments.

**Fig. 5:**
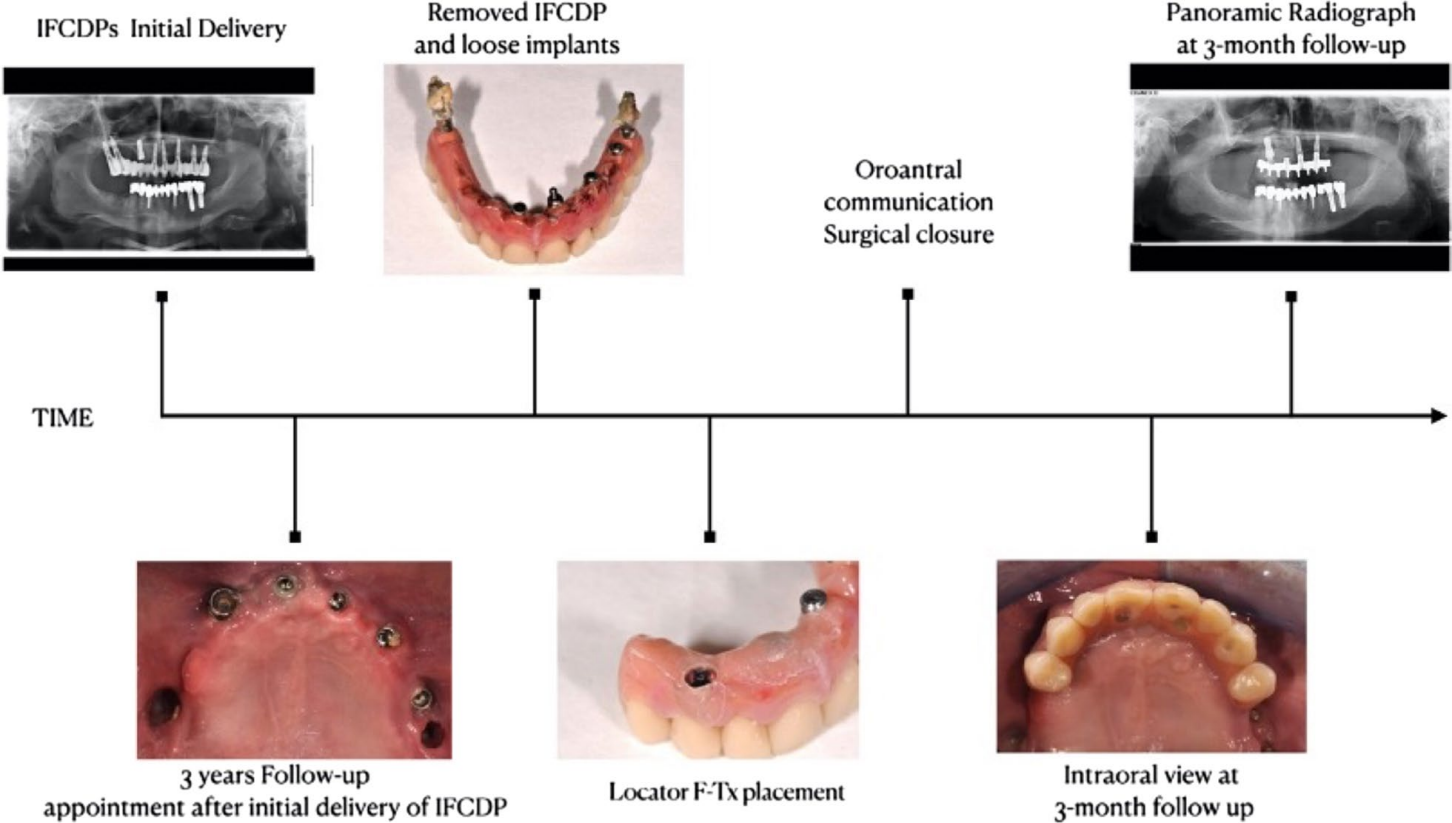
A schematic of the treatment timeline

After the initial post-insertion adjustments, the follow-up visits were scheduled. The first appointment was 1 week after the prosthesis delivery and the subsequent follow-up appointments were 1, 3, and 6 months after the delivery. During the 3-month appointment, the black processing attachment was replaced by the blue low-retention attachment (Fig. 4c). During the follow-up period, no biological or technical complications were observed. The treatment timeline is displayed in Fig. 5.

## Discussion

The failure of multiple implants in IFCDPs commonly requires a new treatment plan to restore function and aesthetics. [5, 8] Minimally invasive treatment approaches are desirable with severely medically compromised patients [6]. The present report described the use of a novel attachment system in a medically compromised patient. Published reports on the use of this system for medically compromised patients are missing, and only two publications report the use of the system in two healthy patients. [5, 9]

In the present case, bringing the patient back to his initial situation with the IFCDP on seven healthy implants would have required extensive surgical procedures including bone augmentation and implant placement. Even though not contraindicated [2, 10, 11] these procedures present with higher morbidity and may lead to complications, especially considering the patient’s prior interventions, age, and comorbidities, which included a history of lung embolism, heart-acquired valvulopathy with an artificial biological valve, advanced diabetes, high blood pressure, and permanent anticoagulation therapy. These factors contributed to the decision not to perform further interventions.

Implant survival rates in patients with cardiovascular diseases and antihypertensive therapy are similar to those of healthy patients [2]. For diabetes patients, when the disease is not well-controlled (hemoglobin A1c ≥ 8.0%), it can have a negative influence on implant survival rates, with reported ranges varying from 86.3% (24 months) to 100% (12 months) [2, 12, 13]. Dental implant placement for patients using oral anticoagulants is not contraindicated, and the discontinuation of the medication is not recommended for implant placement. However, when autogenous bone grafts, extensive flaps, or osteotomy preparations extending outside the ridge envelope are required, consultation with a specialist is recommended [2, 14, 15].

In the present patient’s situation, the clinician faced the challenge of providing a time-efficient, immediate solution. However, the angulation-compensating attachment system enabled the rescue of an existing IFCDP in a single visit. The system allowed the modified IFCDP to fit on unfavorably placed implants. Four to six implants are recommended to support complete-arch fixed prostheses [6]; however, because adjustments were made on the existing prosthesis based on the shortened dental arch concept, and the attachment system described herein was used, the delivery of a fixed prosthesis was possible. Fixed shortened dental arch-prostheses are effective because they minimize the risk of biological complications by avoiding additional implant placement or bone augmentation procedures [6–8].

The attachment system and its mechanism should be carefully studied by clinicians before its use, as the system is unique in how it functions. The insertion and removal of the system is also unique, and removal requires a special tool which should be used by the clinician for correct removal [5, 16].

## Conclusions

The demonstrated angulation-compensating fixed attachment can be used when the immediate adjustment of a fixed screw-retained IFCDP is required due to implant loss. The angular and rotational freedom of the attachment system enables the use of this technique even when the implants are prosthetically unfavorably aligned. Nevertheless, further clinical studies are needed in order to assess the attachment system’s long-term outcomes.

## Data Availability

Raw data of taken pictures is available.

## Abbreviations

IFCDP: Implant-supported fixed complete dental prosthesis
PMMA: Poly(methyl methacrylate)
HbA1c: Glycated hemoglobin
